# Submissive behaviour is affected by group size in a social fish

**DOI:** 10.1098/rsos.240539

**Published:** 2024-05-29

**Authors:** Chelsie Hirons-Major, Tommaso Ruberto, William T. Swaney, Adam R. Reddon

**Affiliations:** ^1^ School of Biological and Environmental Sciences, Liverpool John Moores University, Liverpool, UK

**Keywords:** aggression, cooperative breeding, daffodil cichlid, group living, *Neolamprologus pulcher*, submission

## Abstract

For social groups to form and be stable over time, animals must develop strategies to cope with conflict among group members. Animals may behave submissively either by fleeing from an aggressor, or by signalling submission. The use of these two submissive responses may vary depending on the social and ecological context. Group size is a key aspect of social context for group living animals, as individuals in smaller groups may respond to aggression differently than those from larger groups. Here, we examine the relationship between group size and submissive behaviour in a cooperatively breeding fish, the daffodil cichlid (*Neolamprologus pulcher*). We found that subordinate fish showed similar levels of submission signals in response to dominant aggression in larger and smaller groups, however, subordinates from larger groups were less likely to flee from dominant aggression than those in smaller groups. Subordinates in larger groups also showed more digging behaviour which may be also used to avoid conflict with the dominant group members. Our data show that social context affects submissive behaviour in a cooperatively breeding fish.

## 1. Introduction

Living in a social group yields several benefits, such as increased foraging success [1], better defence against predators [2] and greater access to reproductive partners [3]. Conversely, living in a group may also engender conflict over resources such as food [4] or mating opportunities [5]. Social conflict is likely to be costly for the individual regardless of the outcome (e.g. [6,7], but see [8]) owing to the risk of injury, and the trade-off in time and energy with other activities necessary for survival such as foraging or territory defence [9]. For group living to be stable, animals must employ strategies to mediate the costs of conflict [10].

An individual may avoid the costs of receiving aggression by behaving submissively, either by fleeing from the aggressor or by signalling submission to appease the aggressor and reduce the risk of escalation [11,12]. The tendency for a subordinate animal to behave submissively may depend on both internal and external factors. For example, female Syrian hamsters (*Mesocricetus auratus*) whose levels of circulating gonadal testosterone have been experimentally increased are significantly less likely to show submissive behaviour when placed with an intruder than hamsters in the control group [13]. Veiled chameleons (*Chamaeleo calyptratus*) that face highly aggressive opponents during contests are more likely to darken their colouring, signalling submission [14]. The type of submissive behaviour shown may depend on the ecological context in which the interaction takes place. For example, when the local predation risk is high, it may be better to appease aggressors through submission signals than risk flight in a dangerous environment [15].

The social context may also affect how animals react to conflict. For example, animals living within a permanent social group may have greater incentive to reduce the costs of agonistic interactions owing to relatedness [16] or shared interest in maximizing the productivity of the group [17], incentivizing the use of submission signals to avoid escalated interactions [12]. The need to remain near other members of a social group may reduce the tendency to flee long distances [14,18]. Within species, between-group variation in social composition may affect the use of different social strategies, for example, larger groups may contain more bystanders who could eavesdrop on social signals [19,20]. Larger groups may also be more valuable for current members than smaller groups [21], which could affect the motivation to remain well integrated into the group.

Daffodil cichlids (*Neolamprologus pulcher*) are a lamprologine cichlid fish endemic to Lake Tanganyika, Africa. In the wild, they can be found living in social groups made up of 3–20 mixed-sex individuals [5,22]. Groups are organized into linear dominance hierarchies where rank is determined by body size [23] and the largest male and female generally monopolize reproduction within the group [24]. The breeding pair reinforce their status in the group through threat displays and overt aggression, with dominance interactions between dyads concentrated at the top of the hierarchy near to the valuable breeding positions [23]. Subordinate group members temporarily forego their own reproduction while helping the dominant breeders raise their offspring, by investing in tasks such as brood care, territory maintenance and territory defence [25]. The highest-ranking subordinates may become dominant by usurping dominant status, by inheriting status through removal of a dominant individual in a predation event, or by dispersing to another group to take over a breeding position [26,27]. The frequency of intragroup aggression is high, with conflict arising over rank, access to resources and workload [10,28]. Subordinate individuals may respond to aggression by rapidly swimming a short distance away from their attacker (fleeing) or signalling submission [10,29,30].

Group size varies significantly within and across daffodil cichlid populations [5,31,32], and the number of members of a social group has many important consequences, for example, it influences the longevity [30] and reproductive output of the group [5], and the level of workload reduction for the dominants as a result of subordinate help [33,34]. Subordinates in larger groups may show more submission signals compared with smaller groups to maximize tolerance from the breeding pair in a more valuable social group [34]. In the wild, group size is correlated with territory quality, and removing shelters leads to subordinates being evicted and a decrease in group size [5]. To examine the relationship between group size and submissive behaviour, we observed laboratory housed groups which varied in group size while holding territory quality constant. We predict that subordinates in larger groups will show more submission signals and less fleeing behaviour than those in smaller groups. Subordinates in larger groups may also show more digging behaviours compared with smaller groups as territory maintenance behaviour can also be used to appease dominants and avoid aggression in daffodil cichlids [35].

## 2. Methods

### 2.1. Experimental subjects and housing conditions

The subjects used in our study were laboratory reared descendants of daffodil cichlids (*N. pulcher*) caught near Kasakalawe point in Lake Tanganyika, Republic of Zambia. All fish used in this study were born in laboratory housed social groups and remained in their natal group until reaching approximately 2 cm standard length, at which point they were transferred to stock housing aquaria. We analysed previously coded behaviour from 26 breeding groups (14 larger and 12 smaller groups) assembled and observed for previous studies. Smaller groups comprised a dominant breeding pair (breeder male, breeder female) and two non-breeding subordinates (subordinate 1, subordinate 2, ranked based on body size) of unknown sex, while larger groups were made up of a breeding pair (breeder male, breeder female) and 4–6 non-breeding subordinates of unknown sex, of which the largest two (subordinate 1, subordinate 2) were observed. Subordinates were not sexed because most were too small to determine sex by external morphology at the time of group formation. These group sizes are within the range of naturally occurring groups in the wild [5,30] and of laboratory housed groups that have been used in previous studies [23,36–38].

All fish were measured for standard length (from the tip of the snout to the end of the caudal peduncle) prior to group formation (mean SL ± s.e.m.: dominant male = 55.03 ± 1.08 mm; dominant female = 50.57 ± 1.29 mm; subordinate 1 = 37.29 ± 1.15 mm; subordinate 2 = 32.40 ± 1.10 mm). Dominant fish did not differ in standard length between the larger and smaller groups (*t*-tests: males: *t*_23.9_=0.333, *p* = 0.742; females: *t*_20.0_=0.185, *p* = 0.855), however, subordinates did differ in size: both subordinate 1 fish (*t*‐test: *t*_24.0_=2.107, *p* = 0.046) and subordinate 2 fish (*t*‐test: *t*_23.5_=4.845, *p* < 0.001) were larger in the larger groups (subordinate 1: 39.4 ± 1.58 mm; subordinate 2: 36.5 ± 1.15 mm) than in the smaller groups (subordinate 1: 34.9 ± 1.44 mm; subordinate 2: 28.4 ± 1.23 mm). Social groups were housed in 90 l aquaria (56 cm × 43 cm × 38 cm) which were equipped with a heater, a foam filter, a thermometer and approximately 3 cm of fine coral sand. Each aquarium was also furnished with four terracotta caves for use as shelters for all fish and breeding substrate for the dominants, as well as two floating translucent green PET bottles attached near the surface of the water, providing additional refuge for subordinates. Water temperature was maintained at 27 ± 1°C and natural lighting conditions were simulated using a 12:12 h light:dark cycle, with a 15 min gradual lighting transition simulating dusk and dawn. Fish were fed a variety of dried prepared cichlid food daily. Groups were initially assembled by adding subordinate-sized fish from our laboratory stock aquaria into the group housing aquaria, and then 24 h later adding a breeding pair, who were matched in size such that the breeder male was ~5–10 mm longer than the female. During the first week after group formation, subordinate-sized fish may be excluded from the group, and occasionally, the breeding pair show excessive aggression towards one another. We carefully monitored all groups during this initial phase and all excluded subordinates (who exclusively swim near the water surface and are attacked if they approach any of the caves) were removed and returned to stock aquaria. If the number of subordinates dropped below our desired group sizes or the breeders were constantly aggressive towards each other, then the provisional group was dissolved, the remaining fish returned to our laboratory stock, and a new group formed with new fish. This occurred in approximately 20% of attempts to form new groups regardless of group size. All groups included in the study had been stable with all members tolerated in at least some of the shelters and showing aggression levels typical for long-term laboratory housed groups [23] for at least one month prior to the onset of observations.

### 2.2. Behavioural observations and measures

We observed the two largest subordinates in each group at least four times (range 4–10 observations) for 30 min each time, resulting in between 2 and 5 h of scored behaviour per group. Observations took place between 9:00 and 16:00 with no more than one observation per group per day spread across a two week period. Trained observers (T.R.: 9 smaller and 10 larger groups; C.H.-M.: 3 smaller and 4 larger groups) watched the focal groups live and recorded all agonistic interactions between the dominant breeders and focal subordinates. Prior to the observation, the observer sat motionless approximately 1.5 m in front of the aquarium for 10 min to allow the fish to habituate to the observer’s presence.

We scored all aggression directed by dominant breeders to either of the two largest subordinates including overt aggression (chases and bites) and threat displays (fin spreads, head down displays and opercular flares). We examined whether there was a difference in the proportion of overt aggression versus threat displays from dominants in the larger groups compared with the smaller groups using a subset of the sampled groups for which we had this granular data recorded (i.e. observations taken by T.R.: 10 larger groups and 9 smaller groups).

We scored all submission signals shown by the focal subordinates towards dominant breeders where the focal fish tilts their body upward in the water column and/or rapidly quivers their tail. We scored all instances of fleeing behaviour in the focal subordinates in response to aggression from either member of the dominant pair, wherein the focal subordinate rapidly swims away from the breeder for a distance of at least two body lengths towards a shelter or into the water column (for further detail on the agonistic behaviours of daffodil cichlids, see [29]). In response to each instance of aggression received, a subordinate could show one or more instance of fleeing and/or submission signal.

Helping behaviour may also be used to avoid breeder aggression in this species [39,40], so we scored each subordinate’s territory maintenance effort in the form of digging behaviour. Digging behaviour was recorded when the focal fish picked up sand from within or around one of the cave shelters with its mouth and moved the sand at least one body length before redepositing it. Digging behaviour is one of the key aspects of territory maintenance behaviour in this species and may be exhibited as a form of payment by subordinates in exchange for breeder tolerance within the group [39,41].

We examined the number of submission signals shown per aggression received and the number of flees performed per aggression received for each focal subordinate by dividing the number of submission signals or flees, by the total aggression received. All behaviour was summed across the observations for each group (4–10) and all analysed measures were rates either of behaviours per unit time, or per aggression received from dominants, thereby accounting for the variation in observation time across groups.

### 2.3. Statistical analysis

All statistical analysis was conducted using R 4.2.3 [42] and RStudio v. 2023.03.0. We examined the effects of group size, subordinate rank and their interaction on the behaviours of the two highest ranked subordinates in our social groups. There was no difference in the proportion of overt aggression to threat displays across group sizes (*t*_16.7_ = 1.37, *p* = 0.19), so for all subsequent analyses, we summed all aggression into a single score. We fitted separate linear mixed models to the following response measures: aggression received from the dominant pair per hour, submissions per aggression received, flees per aggression received and digging behaviour per minute, using the ‘lme4’ R package [43]. Owing to the difference in size of subordinates between the larger and smaller groups, we included standard length of each subordinate fish as a continuous covariate to account for potential effects of size on each response variable. Group identity was included as a random effect in each model to account for the non-independence of subordinates 1 and 2 in the same social group. We ran Wald type 3 chi-square tests to evaluate the main effects and interactions in each model using the ‘car’ R package [44]. Model fit was evaluated using the ‘performance’ R package [45] by visual inspection of plots to check for linearity, homogeneity of variance, normality of residuals and presence of influential outliers. Data were transformed to meet model assumptions: data on aggression received per hour were square root transformed, data on submissions per aggression and digging per minute were rank transformed, data on flees per aggression were log transformed. Data and R code for all analyses are available on Zenodo [46].

### 2.4. Ethical statement

Animal housing and handling protocols were approved by the Liverpool John Moores University Animal Welfare and Ethics Steering Group (approval number: AR_TR/2018-4) and adhered to the guidelines of the Animal Behaviour Society and the Association for the Study of Animal Behaviour. All fish were monitored daily for any sign of social exclusion or injury, which did not occur once groups were stabilized prior to observation. All observations were drawn from stable social groups showing species typical levels of agonism [10]. Following observation, all fish remained in their social groups or were returned to stock housing aquaria for use in later studies.

## 3. Results

**Figure 1.**
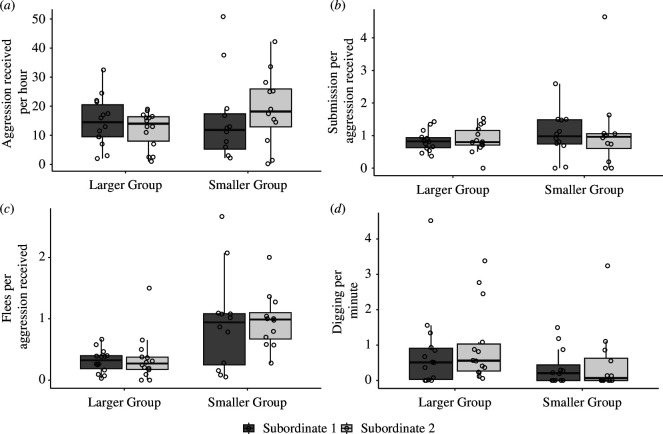
Boxplots with overlaid raw data showing how group size and subordinate rank in social groups of daffodil cichlids affected (*a*) dominants’ aggression, and (*b*) subordinates’ submission signals, (***c***) fleeing and (d) digging. Central lines indicate median values, boxes represent the interquartile range (IQR) and whiskers are 1.5 × IQR. (***a***) Subordinates did not receive different levels of aggression from dominant breeder fish based on group size or subordinate rank. (***b***) Submission by subordinates in response to dominant aggression did not differ between group sizes or subordinate ranks. (***c***) Subordinates fled significantly more often in response to dominant aggression in smaller groups but did not differ between subordinate ranks. (***d***) Subordinates in larger groups engaged in digging behaviour at significantly higher frequency than subordinates in smaller groups but the subordinate ranks did not differ.

There was no effect of group size (χ^2^ = 1.756, d.f. = 1, *p* = 0.185) or of subordinate rank (χ^2^ = 0.486, d.f. = 1, *p* = 0.486) on aggression received from the dominant breeders by subordinates (figure 1*a*), no interaction between group size and subordinate rank (χ^2^ = 1.647, d.f. = 1, *p* = 0.199) and no effect of standard length (χ^2^ = 1.564, *p* = 0.211).

There was no effect of group size (χ^2^ = 0.253, d.f. = 1, *p* = 0.615) or of subordinate rank (χ^2^ = 0.076, d.f. = 1, *p* = 0.783) on submission per aggression received (figure 1*b*), no interaction between group size and subordinate rank (χ^2^ = 0.471, d.f. = 1, *p* = 0.493) and no effect of standard length (χ^2^ = 0.057, *p* = 0.812).

There was a significant effect of group size on flees per aggression received (χ^2^ = 11.436, d.f. = 1, *p* < 0.001, figure 1*c*), as fleeing was more frequent in subordinates from smaller groups (0.950 ± 0.129) than in subordinates from larger groups (0.332 ± 0.055). There was no effect of subordinate rank on flees per aggression (χ^2^ = 0.090, d.f. = 1, *p* = 0.764), no interaction between group size and subordinate rank (χ^2^ = 0.130, d.f. = 1, *p* = 0.718) and no effect of standard length (χ^2^ = 0.138, *p* = 0.710).

Subordinates’ digging behaviour was significantly affected by group size (χ^2^ = 5.509, d.f. = 1, *p* = 0.019, figure 1*d*), as subordinates in larger groups did more digging per minute (0.902 ± 0.210) than subordinates in smaller groups (0.440 ± 0.152). There was no effect of subordinate rank (χ^2^= 0.009, d.f. = 1, *p* = 0.924), no interaction between rank and group size (χ^2^ = 0.871, d.f. = 1, *p* = 0.351) and no effect of standard length (χ^2^ = 0.397, *p* = 0.529) on frequency of digging behaviour.

## 4. Discussion

In this study, we sought to understand how group size may influence submissive behaviour in daffodil cichlid subordinates receiving aggression from the dominant breeders. The aggression received by the focal subordinates from the dominants was consistent across group sizes as was the amount of submission signals shown by subordinates in response. Subordinates in larger groups were less likely to flee from their aggressor than those in smaller groups. Subordinates in larger groups also showed higher levels of territory maintenance, exhibiting more digging behaviour than subordinates in smaller groups.

Across taxa, aggression and eviction rates tend to be higher in smaller groups (fish; birds: [47]; mammals: [47,48], but see [49]). However, we found that aggression received from the dominant breeders was consistent across group sizes. Aggression may not always scale with group size because submission signals may be used as a form of pre-emptive appeasement. Daffodil cichlids pre-empt punishment from dominants by increasing submission signals [28,35]. Daffodil cichlids that show more submission signals receive less aggression from breeders [28] and are more tolerated near the centre of the territory [50]. However, we did not find differences in submission signalling between group sizes.

Subordinates may also behave submissively by fleeing. For this to be effective, the receiver of aggression may need a viable space within the territory to flee to [11]. Subordinates in larger groups had less *per capita* shelter availability in the form of caves and floating shelters, which could have reduced their tendency to flee. Consistent with this, subordinates in larger groups were less likely to show fleeing behaviour in our sample. However, a recent study [51] found that subordinates showed less fleeing when shelter and refuge availability was experimentally increased within groups of standard size (two dominants and two subordinates), suggesting that shelter number is not a limitation on fleeing behaviour in daffodil cichlid groups.

Help is often provided according to need, as seen in the paper wasp (*Polistes dominulus*) where helpers reduce foraging effort when food provisioning is experimentally increased [52]. It is reasonable to assume that *per capita* workload would decrease with increasing group size, since additional helpers would create a load-lightening effect [53], as is found in Kalahari meerkats (*Suricata suricatta* [54]). Breeder workload is also reduced among cooperatively breeding lamprologine cichlids [32,33,55]. We found greater work effort among subordinates in larger groups which may be explained by greater willingness to invest in and/or desire to be tolerated in a larger group. In the wild, daffodil cichlid groups who live in sandier habitats have more subordinates that dig more often, compared with groups living in areas with less sand [22]. Dominant breeders will punish subordinates prevented from digging, and therefore helping may be crucial to acceptance within the group [40]. Although we did not find an effect of body size on digging behaviour, a previous study found that larger helpers dig more [56], and the focal subordinates were larger in our larger groups. Modelling behavioural negotiation in cooperative breeders predicts that helping may be used to pre-emptively appease dominant breeders and keep aggression levels at equilibrium across contexts, fitting with our results [52]. Helpers in larger groups may be more readily replaced by lower ranking individuals than helpers in smaller groups, and therefore may be less valued by the dominant breeding pair [57,58]. This may lead to subordinates increasing their workloads to avoid aggression from the breeding pair in larger groups.

## 5. Conclusions

We found that the submissive behaviour of subordinate daffodil cichlids is influenced by the size of the social group. Subordinates across group sizes showed similar levels of submission signals but subordinates in smaller groups showed more fleeing behaviour than those in larger groups. Subordinates in larger groups also showed more territory maintenance behaviour, which may be used as a form of pre-emptive appeasement of dominants. Our results indicate that social context in the form of group size affects the strategies that social animals use to avoid aggression and mitigate conflict.

## Data Availability

Data and R code for all analyses are available at Zenodo [46].
